# Comparative analysis of *Ligusticum chuanxiong* from Gansu and Sichuan using the fingerprint technique and HS-SPME-GC-MS combined with chemometric analysis

**DOI:** 10.1371/journal.pone.0347839

**Published:** 2026-04-30

**Authors:** Jialing Zhang, Juanjuan Liu, Yiyang Chen, Liangcai Wang, Ke Li, Gonghan Tu, Xiaohui Ma, Ling Jin

**Affiliations:** 1 School of Pharmacy, Gansu University of Chinese Medicine, Lanzhou, Gansu, China; 2 Northwest Chinese-Tibetan Medicine Collaborative Innovation Center, Lanzhou, Gansu, China; 3 Long-yao Industry Innovation Research Institute, Lanzhou, Gansu, China; 4 Gansu Engineering Research Center for Evaluation, Protection and Utilization of Rare Traditional Chinese Medicine Resources, Lanzhou, Gansu, China; Yantai Institute of Technology, CHINA

## Abstract

*Ligusticum chuanxiong* (LC) is an important medicinal herb widely used in traditional Chinese medicine (TCM). The variety cultivated in Gansu Province, known as *chuanxiong* (*Xixiong,* LX), exhibits distinct morphological characteristics compared with LC from other regions. In this study, we performed a comprehensive comparative analysis of LC and LX. Odor and color were quantified using an electronic nose and a colorimeter, respectively. Volatile components were profiled by headspace solid-phase microextraction coupled with gas chromatography-mass spectrometry (HS-SPME-GC-MS), while high-performance liquid chromatography (HPLC) was used to quantify chemical constituents and establish chromatographic fingerprint profiles. Pearson correlation analysis was further conducted to elucidate the relationships among chromaticity parameters, odor and other phenotypic traits, and chemical constituents, as well as their associations with altitude. Results showed that LX had significantly greater dry weight, diameter, and other morphological parameters than LC. LX powder displayed a brown hue, whereas LC powder appeared yellowish-brown. LC exhibited higher response values for the W1S and W2S sensors compared with LX. HPLC analysis revealed that the contents of ligustilide (LI), senkyunolide I (SI), senkyunolide H (SH), and 3-butylphthalide (3B) were significantly higher in LC than in LX (*p* < 0.01), whereas the senkyunolide (SA) was significantly lower in LC (*p* < 0.05). HS-SPME-GC-MS detected 82 compounds in LX and 62 compounds in LC, with 34 compounds shared between them. Significant correlations were observed among altitude, chromaticity, odor, and chemical composition. This study provides a systematic characterization of the differences between the collected LC and LX, offering insights that may enhance the market competitiveness of LX, promote optimal utilization of regional resources, and support the sustainable development of the LX industry in Gansu Province.

## 1. Introduction

*Ligusticum chuanxiong* (LC) is the dried rhizome of the Umbelliferae plant *Ligusticum chuanxiong* Hort.. It was first recorded in the *Shennong’s Classic of Materia Medica* (*Shennong Ben Cao Jing*) and has long been valued in traditional Chinese medicine (TCM) for its health-promoting properties. Historically, LC has been prescribed for the treatment of a variety of disorders, including chest pain, dysmenorrhea, abdominal discomfort, headaches, and rheumatic arthralgia [1]. Modern phytochemical studies have identified a wide range of bioactive constituents in LC, including alkaloids, volatile oils, polysaccharides, and phenolic acids [2–8]. At present, LC is primarily obtained from cultivated sources, with its core production areas concentrated in Dujiangyan, Pengzhou, and Meishan in Sichuan Province, China. Historical accounts in the *Compendium of Materia Medica* (*Bencao Gangmu Shiyi)* indicate that the herb was once cultivated commercially in multiple provinces, including Gansu, Yunnan, Jilin, and Jiangxi. In Gansu Province, Xixiong (LX) refers to LC cultivated locally. According to the 2009 edition of the *Standard of Chinese Herbal Medicine in Gansu Province*, LX was introduced from Sichuan and is now mainly produced in Longnan and Huating [9].

Classical literature such as the *Revised Compendium on Counterfeit Drugs* (*Zengding Weiyao Tiaobian*) records morphological differences between LC and LX, while the *Supplement to the Compendium of Materia Medica* and the *Enlightened Primer of Materia Medica* (*Bencao Mengquan*) note distinctions in their pharmacological activities and medicinal applications. Recent studies have demonstrated significant regional variability in the composition and content of volatile oils in LC [10]. In terms of total volatile oil content, the ranking follows Sichuan > Jiangsu > Gansu > Yunnan. Notably, senkyunolide I (SI) content can vary by up to threefold among regions, and ligustilide (LI) content by up to fourfold [11–13]. Furthermore, LX contains significantly higher levels of ferulic acid (FA) and alkaloids compared to Sichuan-produced LC [14,15]. These quality differences may be attributed to factors such as differential gene expression [16], cadmium stress responses [17], and environmental influences including altitude [18].

However, the apparent morphological differences between LX and LC have hindered LX’s market competitiveness, often attracting increased regulatory scrutiny and reducing growers’ willingness to cultivate it. To address these challenges, the present study undertakes a systematic comparative analysis of LC and LX. Intelligent sensory technologyies including colorimeter and Ean electronic nose, are applied to quantitatively evaluate chromatic and olfactory characteristics. Advanced analytical techniques, such as headspace solid-phase microextraction coupled with gas chromatography-mass Spectrometry (HS-SPME-GC-MS) and high performance liquid chromatography (HPLC), are eused to characterize their chemical profiles and establish chemical fingerprints. Multivariate statistical analyses, including principal component analysis (PCA) and orthogonal partial least squares-discriminant analysis (OPLS-DA), are then employed to clarify compositional differences. Finally, correlation analyses are performed to investigate the intrinsic relationships among altitude, chromaticity, odor, and chemical composition. The outcomes of this study are expected to provide a scientific basis for understanding quality differences between LC and LX, and to support the development and quality control of LX derived medicinal materials.

## 2. Materials and methods

### 2.1. Sample source

Ten batches each of LC and LX samples were authenticated by Professor Ling Jin of Gansu University of Chinese Medicine, as detailed in S1 Table in S2 File. All LC and LX samples have were deposited at the Longyao Industry Innovation Research Institute.

### 2.2. Reagents

Reference standards, including chlorogenic acid (CGA), tetramethylpyrazine (TMP), FA, SI, senkyunolide H (SH), senkyunolide A (SA), coniferyl ferulate (CF), LI, 3-butylidenephthalide (3B) and 3-n-butylphthalide (3nB) (purity ≥ 98%), were purchased from Shanghai Yuanye Biotechnology Co., Ltd (Shanghai, China). Analytical-grade glacial acetic acid (Tianjin Damao Chemical Reagent Factory, Tianjin, China), 2-Octanol (CATO Research Chemicals Inc., Guangzhou, China), sodium chloride (Tianjin Damao Chemical Reagent Factory, Tianjin, China), chromatographic-grade acetonitrile (Tianjin Starmark Technology Development Co., Ltd., Tianjin, China) and Wahaha bottled drinking water (Hangzhou Wahaha Group Co., Ltd, Hangzhou, China) were useed for analyses.

### 2.3. Determination of appearance traits

The traditional empirical identification method was applied to characterize the morphological features of LC and LX by examining their external attributes, including morphology, size, color, texture, cross-sectional characteristics, and odor. The diameter (measured at the thickest point of the mid-section) and length (from rhizome apex to base) of each sample were measured using an electronic vernier caliper (Sanliang Corporation, Jiangsu, China). Dry weights were determined with an EX224ZH electronic balance (Aohaus Instruments Changzhou Co., Ltd. Changzhou, China), with each specimen weighed in ten replicates to obtain a mean value. Subsequently, the length-to-diameter ratio was calculated as length divided by diameter for a more comprehensive morphological assessment.

### 2.4. Chromaticity measurement

Chromaticity was determined using a high-precision colorimeter NH310 (Shenzhen ThreeNH Technology Co., Ltd., Shenzhen, China) equipped with a D65 light source, a 5 mm measurement aperture, and a 10° observer angle. Prior to measurement, the instrument was was warmed up for 5 min and calibrated against a standard white calibration plate, with baseline values recorded as L0, a0, and b0. Approximately 2.0 g of LC and LX (passed through a No. 4 sieve) was evenly spread over the measurement port. The chromaticity parameters, including lightness (L*), red-green value (a*), and yellow-blue value (b*), were recorded. Nine parallel measurements were performed for each sample, and the average values were calculated. The total color difference (ΔE) was computed according to the following equation: ΔE = (ΔL*² + Δa*² + Δb*²)^1/2^, where ΔL* = L* - L_0_, Δa* = a* - a_0_, and Δb* = b* - b_0_ [19].

### 2.5. Electronic nose analysis

The odor profiles of the samples were analyzed using a Pen3 electronic nose (Airsense, Schwerin, Germany). Detailed information on the sensor array is provided in S2 Table in S2 File [20]. To optimize the measurement conditions, four influencing factors were initially evaluated: sample particle size (passed through No.2, 3, 4, 5 sieves), electronic nose air intake flow rate (100, 150, 200, 250, 300 mL·min ⁻ ¹), sealing time prior to measurement (5, 10, 15, 20, 25 min) and sample weight (0.5, 1.0, 1.5, 2.0, 2.5 g). During measurement, the injection needle was directly inserted into a 15 mL headspace vial containing the sample powder at room temperature. The sampling duration was 150 s with a 1 s acquisition interval. The sensor array cleaning time was set to 60 s. The response value of each sensor to the sample was recorded for analysis.

### 2.6. Determination of components by HPLC and establishment of LC and LX fingerprints

Quantitative determination of components was carried out according to the method described in reference [21], using an LC-2023C-Plus HPLC (Shimadzu Corporation of Japan, Kyoto, Japan). Chromatographic separation was achieved using a Zafex Supperfex RP-C18 column (250 × 4.6 mm, 5μm, Shandong Zhefen Scientific Instrument Co., Ltd., Shandong, China). The mobile consisted of acetonitrile (solvent A) and 0.5% acid solution (solvent B), with the following gradient program: 0−15 min, 10%−16% A, 15−30 min, 16%−30% A, 30−35 min, 30%−40% A, 35−70 min, 40%−55% A, 70−75 min, 55%−25% A, 75−80 min, 25%−10% A. The flow rate was 1.0 mL·min ⁻ ¹, detection wavelength 285 nm, column temperature 30 °C, and injection volume 10 μL. For sample preparation, 1.0 g of LC and LX powder was placed in a 50 mL Erlenmeyer flask with 20mL of 75% ethanol refluxed for 30 min, cooled to room temperature, and adjusted for any weight loss with 75% ethanol. The extract was filtrate and concentrated to a final volume of 5.0 mL in a volumetric flask, and subsequently filtered through a 0.45 μm microporous membrane prior to injection.

Standard stock solutions of CGA, TMP, FA, SI, SH, SA, CF, LI, 3B and 3nB were prepared in methanol at concentrations of 1.225, 0.0397, 0.736, 0.788, 0.03379, 7.2673, 0.2468, 5.68, 3.375 and 0.3764 mg·mL ⁻ ¹, respectively. Calibration curve were established by plotting peak area (Y) against mass concentration (X)for each analyte. The resulting regression equations and correlation coefficients (R^2^) are listed in S3 Table in S2 File.

### 2.7. Comparison of volatile components between LC and LX by HS-SPME-GC-MS

Analysis of volatile constituents was performed using a headspace solid-phase microextraction coupled with gas chromatograph–mass spectrometer (HS-SPME-GC-MS, 7890B GC–7000D MS system, Agilent Technology Co., Ltd., Beijing, China) [22]. For each batch, 0.060 g of sample powder was weighed into a 20 mL headspace vial, followed by the addition of 3 mg·L ⁻ ¹ 2-octanol as an internal standard. Saturated sodium chloride solution was added prior to heating at 80 °C for 30 min. The HS-SPME-GC-MS was then inserted into the headspace vial and heated for additional 30 min. Subsequently, the sample was desorbed at 250 °C for 5 min. Separation was performed on an HP-5MS column (30 m × 0.25 mm × 0.25 μm film thickness). The oven temperature program was as follows: initial temperature 50 °C (hold 2 min) increased at 5 °C·min ⁻ ¹ to 180 ℃, then maintained at 250 °C for 5 min. The mass was operated in electron impact mode at 70 eV under full scan mode with a mass range of m/z 40–600. The ion source and quadrupole temperatures were set to 230 ℃ and 150 °C, respectively.

### 2.8. Data analysis

Radar plots, box plots and histograms were generated using Origin 2021 and GraphPad Prism 9.5. Differences between groups were analyzed by one-way analysis of variance (ANOVA), with significance set at *p* < 0.05. Chromatographic fingerprints were generated by inputting peak area data into the Similarity Evaluation System of Chromatographic Fingerprint of TCM (version 2012). Cluster analysis (CA) and factor analysis were performed in SPSS software (version 27.0), whereas PCA and OPLS-DA were conducted using SIMCA 14.1. Correlation heatmaps were constructed via the online platform https://www.chiplot.online.

## 3. Results

### 3.1. Determination of appearance traits

**Fig 1:**
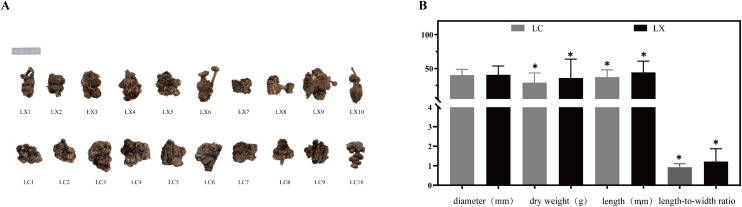
(A) Diagrams of the Traits of LX and LC Medicinal Materials; (B) Determination Results of Trait Indicators of LX and LC(* *p* < 0.05).

The key characteristics for the identification of LC and LX are summarized in S4 Table in S2 File, with corresponding images of the medicinal materials shown in Fig 1A. The measurement data of morphological indicators were analyzed using GraphPad Prism 9.5, resulting in the generation of column charts (Fig 1B). The dry weight (35.87 ± 27.94 g), length (44.38 ± 16.48 mm), and length-to-width ratio (1.22 ± 0.65) of LX were significantly higher than those of LC (29.15 ± 14.24 g, 37.40 ± 10.71 mm, and 0.93 ± 0.18, respectively), with statistically significant differences observed between the groups (*p* < 0.05 for all comparisons). However, the diameter of LX (40.74 ± 12.92 mm) showed only a slight increase compared to LC (40.47 ± 8.37 mm), and no statistically significant difference was observed between the two species.

### 3.2. Chromaticity measurement

**Fig 2:**
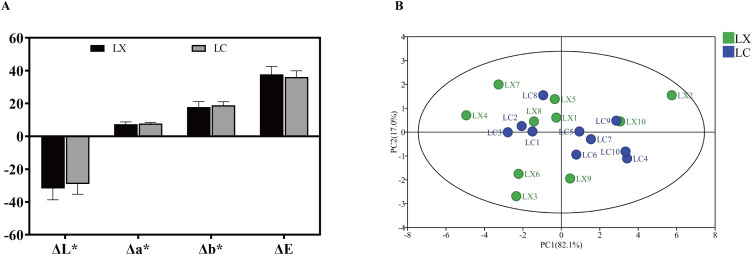
(A) LX and LC color analysis chart; (B) PCA Score Plot of Color Difference between LX and LC.

To minimize measurement errors related to the chromameter, white paper was employed as the standard reference, yielding initial values of L_0_ = 97.12, a_0_ = −0.38, and b_0_ = 0.26. The color ranges of LX powder were recorded as 31.21 ± 1.19 to 45.67 ± 0.63 (L*) and 30.79 ± 2.38 to 41.77 ± 1.69 (LC powder). The measurement data were analyzed using GraphPad Prism 9.5 to generate column charts (Fig 2A). Results indicated that LX powder exhibited higher absolute ΔL* and ΔE values compared to LC powder, while Δa* and Δb* values remained similar between the two powders. This suggests that the brightness of LX powder is low and its color is dark brown, whereas LC powder displayed a high brightness with a light yellow-brown color. PCA revealed two principal components with eigenvalues greater than 1, accounting for 82.1% and 17.0% of the variance, respectively (cumulative contribution rate: 99.1%). Both LC and LX powders showed overlapping distributions in the first and second principal components, indicating that these two components effectively captured the majority of color variation information. This minimal differentiation in principal component space illustrated relatively small color differences between LC and LX powders (Fig 2B).

### 3.3. Electronic nose analysis

#### 3.3.1. Electronic nose response value and factor level screening.

An electronic nose device was utilized to capture the odor profiles of LC and LX samples, yielding response plots from 10 sensors (S1 Fig in S2 File). In S1 Fig in S2 File, the position on the curve (G/G0, where G represents the measured gas value, and G0 denotes the air value) illustrates the variation in relative resistivity throughout the passage of LC and LX odorants across the sensor channel over time. Initially, from the onset of injection to the eventual stabilization of sample gas, G/G0 exhibited a rapid increase, followed by a decline, and eventually reached a stable state. This pattern signifies the sensitivity and consistency of the electronic nose device in detecting the aroma of LC, with the chemical composition present in LC’s aroma being relatively consistent and volatile. The data collected by the electronic nose device were analyzed using SPSS 27.0 software. Investigations were conducted to discern the impact of sample particle size, airflow rate of the electronic nose device, storage duration under sealed conditions, and sample mass on the content of primary constituents (chromatographic peaks exhibiting strong discriminatory ability and substantial peak area). The findings are depicted in S2 Fig in S2 File. The outcomes delineate the optimal operational parameters for the electronic nose device, including a storage duration of 15 minutes, an ideal sample mass of 2.0 g, particle size from the No.3 sieve, and an injection volume of 150 mL/min.

#### 3.3.2. Comparison of odor difference between LC and LX.

**Fig 3:**
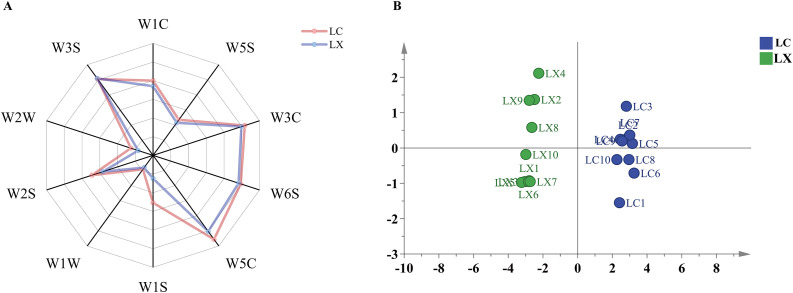
(A) LC and LX sensor response radar chart; (B) PCA of LC and LX electronic nose data.

Utilizing an electronic nose to capture the comprehensive olfactory profile of the sample, simulating the olfactory perception of humans, aids in circumventing the subjective nature of sensory evaluation [23]. Employing the electronic nose, the overall aroma of LC and LX samples was characterized. As illustrated in Fig 3A, Sensors W5C (sensitive to alkane aromatic constituents), W3S (responsive to alkanes), W6S (detects ammonia and water, sensitive to aromatic compounds), W3C (primarily selective to hydrogen), and W2S (sensitive to ethanol) exhibited elevated response values. This indicates that the aroma constituents of LC and LX samples primarily consist of aromatic compounds, alkanes, ammonia substances, alcohols, and hydrogen. Notably, LC exhibited significantly higher response intensities to W5C, W1S, W2S, and W1C sensors compared to LX.

PCA stands as a powerful multivariate statistical technique, facilitating the transformation and dimensionality reduction of complex datasets, while also enabling linear classification of outcomes (Fig 3B). The findings revealed a clear demarcation between LC and LX samples, with the first principal component boasting a variance contribution rate of 82.1%. Additionally, the variance contribution rate of the second principal component amounted to 9.43%, resulting in a cumulative contribution rate of 91.5% (demonstrating the method’s viability, with cumulative PC > 85%). Evidently, the initial two principal components encapsulated the majority of information from the 20 sample batches, thereby serving as a reliable basis for quantitative differentiation between LC and LX.

### 3.4. Establishment of LC, LX fingerprint and determination of chemical composition content

#### 3.4.1. Establishment of HPLC fingerprint.

**Fig 4:**
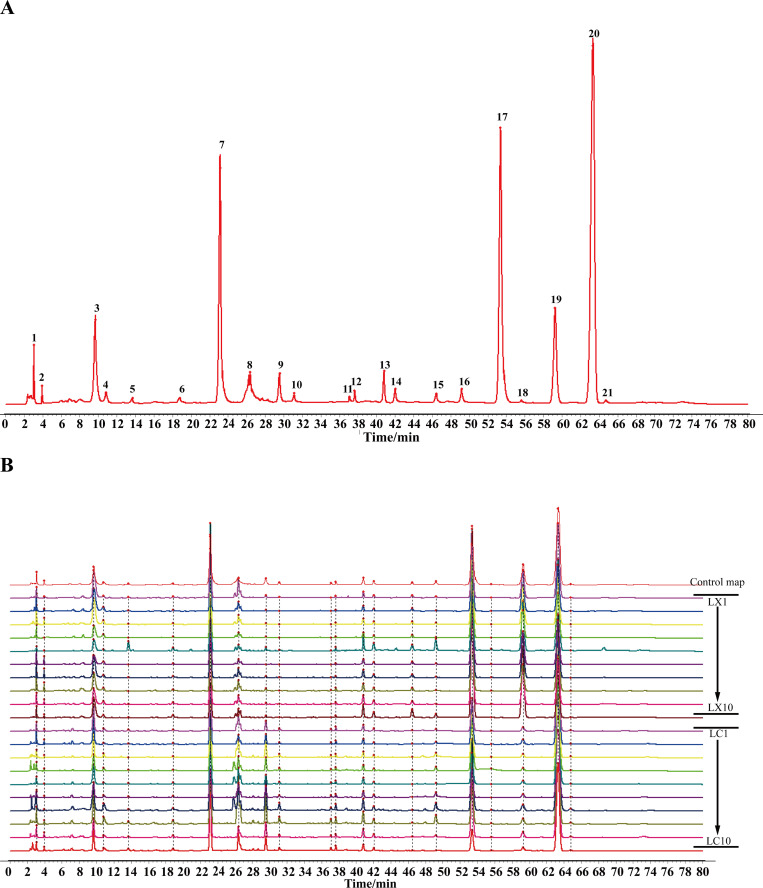
(A) LC and LX control map. (4: chlorogenic acid, 5: tetramethylpyrazine, 7: ferulic acid, 9: senkyunolide I, 10: senkyunolide H, 16: conifery ferulate, 17: senkyunolide A, 18: 3-n-butylphthalide, 20: ligustilide, 21: 3-butenylphthalide); (B) LC and LX HPLC fingerprint.

The precision, sample repeatability, and stability of the instrument were evaluated based on the retention time and peak area of common peaks. The results demonstrated that the relative standard deviation (RSD%) of the retention time for each common peak was less than 2.0%. Subsequently, the chromatographic data from the samples were imported into the Similarity Evaluation System for Chromatographic Fingerprint (SESCF) of TCM. LC1 was designated as the reference chromatogram, with a time window set at 0.1, and control chromatograms were established using the median method (Fig 4A). Subsequently, HPLC fingerprints were constructed for the 20 sample batches (Fig 4B). A total of 21 peaks, which demonstrated robust qualitative results, were identified as common peaks. Through comparison with the chromatogram of the mixed reference solution and consultation of relevant literature, 10 peaks were confidently identified. Notably, peak 4 corresponded to CGA, peak 5 to TMP, peak 7 to FA, peak 9 to SI, peak 10 to SH, peak 16 to CF, peak 17 to SA, peak 18 to 3nB, peak 20 to LI, and peak 21 to 3B.

#### 3.4.2. Similarity evaluation.

The similarity evaluation results, as depicted in S5 Table in S2 File, revealed that the similarity indices ranged from 0.965 to 1 for LC samples and from and from 0.903 to 1 for LX samples, respectively. However, the similarity between LC and LX samples was above 0.612. This indicates that the internal similarity of both LC and LX samples is good, while there are certain quality differences between them.

#### 3.4.3. Cluster analysis.

**Fig 5:**
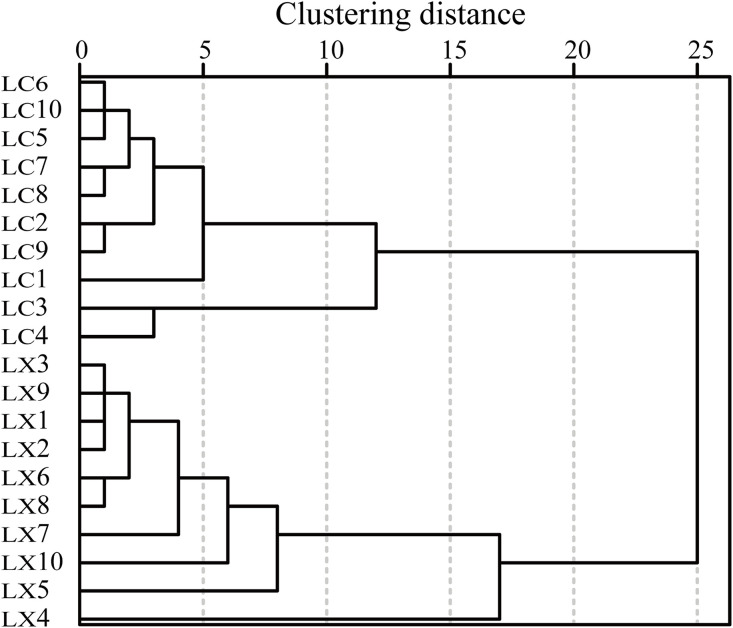
LC and LX cluster analysis tree diagram.

SPSS 27.0 software was employed to conduct cluster analysis on 20 batches of LC and LX samples, using the 21 common peak areas as variables. The inter-group connection metho (IGCM) and square euclidean distance (SED) were adopted as measurement criteria to evaluate the similarity between the sample data. The samples were classified based on their similarity (see Fig 5). When the cluster spacing was set to 25, the 20 batches of samples could be divided into two categories: LC1 to LC10 and LX1 to LX10, indicating discernible differences between LC and LX. When the clustering spacing was reduced to 15, the samples were further classified into three categories: LC1 to LC10, LX1 to LX3, and LX5 to LX10, with LX4 being categorized separately. This suggests that the intraspecific quality variation of LC was minor, while notable differences existed in the intraspecific quality of LX.

#### 3.4.4. Factor analysis.

The peak areas of 21 common peaks observed in the fingerprints of 20 batches of LC and LX samples were used as variables for factor analysis, which was performed using SPSS 27.0. The extraction criterion employed was set to an eigenvalue > 1, resulting in an initial factor loading matrix containing 4 principal components (S6 Table in S2 File). The eigenvalue and variance contribution rates are presented in Table 1. Notably, the cumulative variance contribution of the four principal components reached 87.644%, indicating that these components accounted for 87.644% of the information derived from the original variables. Therefore, these four principal components could effectively replace the 21 common peaks in the fingerprint analysis for evaluating the quality of LC and LX samples.

**Table 1:** Eigenvalue and variance contribution rate.

Principal component factor	Eigenvalue	Variance contribution rate %	Cumulative variance contribution rate %
1	8.911	42.434	42.434
2	5.645	26.881	69.315
3	2.819	13.426	82.741
4	1.03	4.904	87.644

The factor loading matrix reveals the correlation coefficients between the 4 principal components and the 21 original variables (chromatographic peaks), as illustrated in S7 Table in S2 File. Specifically, the first principal component primarily reflects information from chromatographic peaks 8, 9, 10, 11, 12, 20 and 21; The second principal component is mainly associated with peaks 5, 6, 7, 13, 14 and 16; The third principal component is characterized by peaks 1, 2, 4 and 17; The fourth principal component is predominantly influenced by peak 18.

#### 3.4.5. Orthogonal least squares-discriminant analysis (OPLS-DA).

**Fig 6:**
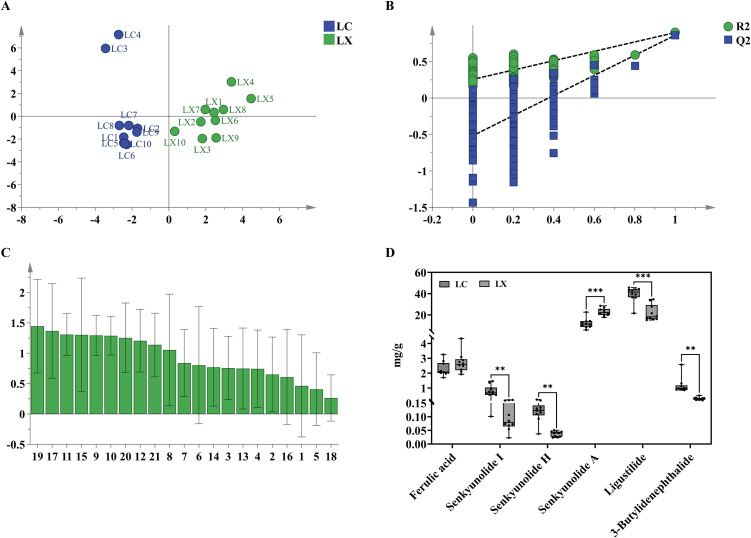
Orthogonal partial least squares discriminant analysis (OPLS-DA) analysis of LC and LX. **(A)** OPLS-DA score and load map; **(B)** Results of 200 cross-validations of OPLS-DA model; **(C)** The OPLS-DA model obtained a histogram with VIP value > 1.0, and a confidence level of 95%; **(D)** Box diagram for the determination of chemical composition content. The significant difference is determined by *p <* 0.05 (*), 0.01 (**), 0.001 (***).

Multivariate statistical analysis was performed using SIMCA 14.1 to conduct OPLS-DA modeling on the 21 common peak areas in the fingerprints of LC and LX samples as variables, aiming to further explore the differences in their chemical compositions. As shown in Fig 6A, the OPLS-DA model yielded an R^2^X value of 0.66, with R^2^Y and Q^2^ values of 0.898 and 0.862, respectively—all exceeding the threshold of 0.5, indicating the model’s stability and reliability. The OPLS-DA score plot demonstrates clear separation of LC and LX samples into two distinct clusters along the Y-axis, consistent with the results of previous analytical methods. The model’s accuracy was validated through 200 permutation tests (Fig 6B). The test parameters, R^2^ = (0, 0.274) and Q^2^ = (0, −0.478), indicate that all R^2^ and Q^2^ values on the left are lower than the rightmost value. Additionally, the regression line of the Q^2^ point intersects the vertical axis (left) below zero, confirming no overfitting in the model and supporting its suitability for discriminant analysis of LC and LX samples. Key marker components contributing to chemical composition differences were identified using variable importance for the projection (VIP). As shown in Fig 6C, the abscissa represents the fingerprint peak numbers, with VIP values exceeding 1 serving as the screening criterion. The VIP variables, ranked by their contribution from highest to lowest, included fingerprint peaks 19, 17, 15, 11, 20, 9, 10, 12, and 21. Among these, five components were verified by reference substances: peak 17 (SA), peak 20 (LI), peak 9 (SI), peak 10 (SH), and peak 21 (3B). Their contributions to the differences followed the order: SA > LI > SI > SH > 3B, highlighting their potential as differential markers for LC and LX.

#### 3.4.6. Determination of chemical constituents by HPLC.

Both the *Chinese Pharmacopoeia* [1] and the *Gansu Provincial Standards for Chinese Medicinal Materials* [9] stipulate a minimum FA content of 0.1% as the criterion for assessing the quality of medicinal substances of LC and LX. Therefore, the FA content, along with that of five other distinct components in LC and LX samples, was precisely quantified using HPLC. The concentrations of these six components were determined based on the regression equation derived from the standard curve. The FA content in LC and LX samples ranged from 0.16% to 0.39% and 0.14% to 0.49%, respectively. These results indicate that the quality of all 20 sample batches met the criteria specified in the *Chinese Pharmacopoeia* [1] and the *Gansu Province’s herbal medicine standard* [9]. Notably, the FA content in LX5, 6, and 7 exceeded 0.25%. Furthermore, the overall FA content in LX was consistently higher than that in LC, aligning with previous research findings [15]. As shown in Fig 6D, the concentrations of LI, SI, SH, and 3B in LC were significantly higher than those in LX (*p* < 0.001, *p* < 0.01, *p* < 0.01, *p* < 0.01, respectively). In contrast, SA was significantly lower in LC compared to LX (*p* < 0.001). These findings corroborate the predictions of the OPLS-DA model. Consequently, LI, SI, SH, 3B and SA emerge as pivotal differential components influencing the quality disparity between LC and LX.

### 3.5. HS-SPME-GC-MS analysis

#### 3.5.1. HS-SPME-GC-MS detection of volatile compounds.

**Fig 7:**
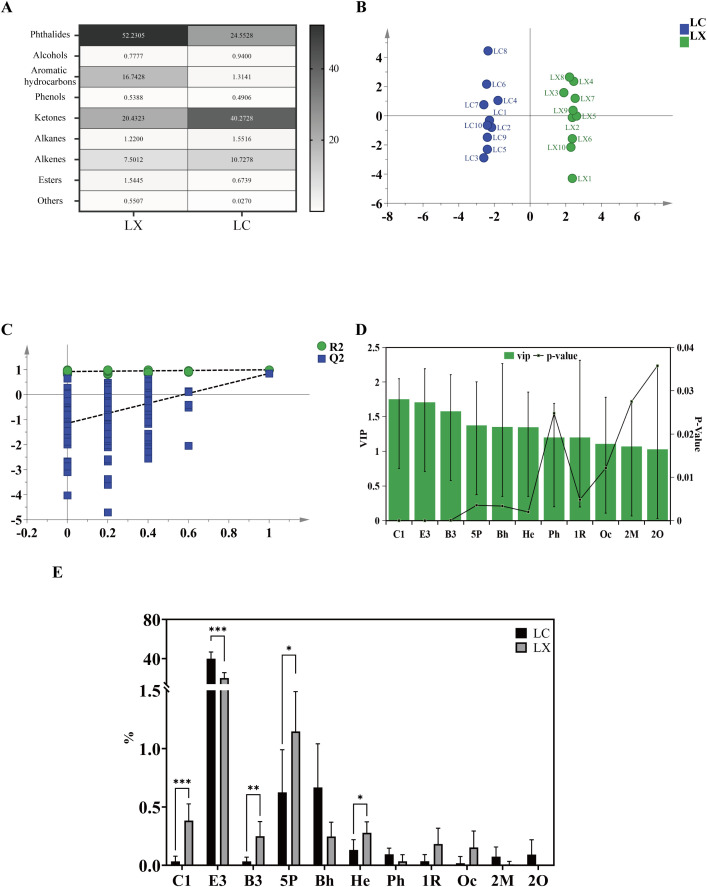
(A) The types of volatile components in LC and LX; (B) OPLS-DA score graph of LC and LX based on HS-SPME-GC-MS; (C) Permutation test results; (D) VIP value > 1, p < 0.05 difference component histogram; (E) The content of 11 different components. (Cyclohexane, 1-methylene-4-(1-methylethenyl)-: C1; (E)-3-Butylidene-4, 5-dihydroisobenzofuran-1(3H)-one: E3; Bicyclo[3.1.1]heptane, 6, 6-dimethyl-2-methylene-(1S)-: B3; 5-Pentylcyclohexa-1, 3-diene: 5P; Bicyclo[3.1.0]hexane, 4-methylene-1-(1-methylethyl)-: Bh; Hexadecanoicacid, methylester: He; Phenol, 5-ethenyl-2-methoxy-: Ph; (1R)-2, 6, 6-Trimethylbicyclo[3.1.1]hept-2-ene: 1R; 12, 15-Octadecadienoic acid, methyl ester: Oc; 2-Methoxy-4-vinylphenol: 2M; 2-Octanol: 2O.) The significant difference is determined by p < 0.05 (*), 0.01 (**), 0.001 (***).

To investigate the composition and content of volatile components in LC and LX, 20 batches of samples were analyzed and identified using HS-SPME-GC-MS to determine their volatile components and relative contents. From S8 Table in S2 File, it can be observed that 110 compounds were identified, among which 62 volatile components were detected in LC, while 82 were identified in LX. Notably, 34 components were common to both groups, whereas LC and LX exhibited 28 and 48 distinctive components, respectively. The volatile component categories in LC and LX are graphically represented in Fig 7A. In LC the order of content for each volatile component category was as follows: ketones > phthalides > alkenes > alkanes > aromatic hydrocarbons > alcohols > esters > phenols. Conversely, in LX, the order differed: phthalides > ketones > aromatic hydrocarbons > alkenes > esters > alkanes > alcohols > phenols. Notably, phthalides, alkenes, and ketones exhibit heightened higher levels in both LC and LX.

#### 3.5.2. Comparison of volatile substances in LC and LX.

OPLS-DA, a supervised multivariate statistical analysis technique, mitigates the influence of uncontrolled variables by integrating predefined categorical variables [24]. In this study, the OPLS-DA discriminant model (Fig 7B) was employed to analyze the variances between LC and LX samples from distinct production locales. In this analysis, the independent variable fitting index (R^2^X) was 0.603, the dependent variable fitting index (R^2^Y) was 0.993, and the model’s predictive index (Q^2^) was 0.5845. Notably, R^2^ and Q^2^ exceeded 0.5, and the proximity of R^2^Y and Q^2^ to 1 indicated acceptable model fitting [25]. Furthermore permutation test results demonstrated that all Q^2^ and R^2^ values (Fig 7C, lower left quadrant) were lower than their original counterparts (Fig 7C, upper right quadrant). To further investigate the differentiation between LC and LX samples, the VIP scores and the p-value from Student’s t-test in the OPLS-DA model were compared (Fig 7D). Using a significance threshold of *p* < 0.05 and a VIP value > 1, eleven distinctive volatile compounds were identified between LC and LX, including 3 olefins, 2 alkanes, 2 esters, 2 phenols, 1 alcohol, and 1 ketone. The relative content differences are illustrated in Fig 7E, which shows that the levels of Cyclohexane, 1-methylene-4-(1-methylethenyl) (C1), Bicyclo[3.1.1]heptane, 6, 6-dimethyl-2-methylene-(1S)- (B3), and 5-Pentylcyclohexa-1, 3-diene (5P), Hexadecanoicacid, methylester (He) in LX were significantly higher than those in LC, whereas the content of E3 was notably lower in LX compared to LC.

### 3.6. Correlation analysis of “Altitude-Chromatic-Odor-Chemical composition”

**Fig 8:**
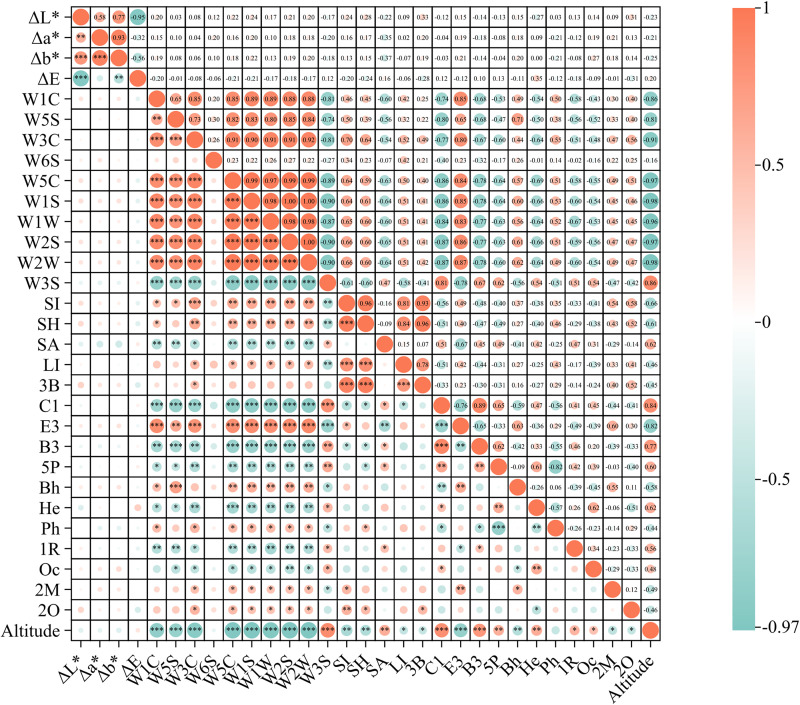
The appearance, odor and differential chemical correlation heat map of LC and LX. (Cyclohexane, 1-methylene-4-(1-methylethenyl)-: C1; **(E)**-3-Butylidene-4, 5-dihydroisobenzofuran-1(3H)-one: E3; Bicyclo[3.1.1]heptane, 6, 6-dimethyl-2-methylene-(1S)-: B3; 5-Pentylcyclohexa-1, 3-diene: 5P; Bicyclo[3.1.0]hexane, 4-methylene-1-(1-methylethyl)-: Bh; Hexadecanoicacid, methylester: He; Phenol, 5-ethenyl-2-methoxy-: Ph; (1R)-2, 6, 6-Trimethylbicyclo[3.1.1]hept-2-ene: 1R; 12, 15-Octadecadienoic acid, methyl ester: Oc; 2-Methoxy-4-vinylphenol: 2M; 2-Octanol: 2O). The significant difference is determined by *p* < 0.05 (*), 0.01 (**), 0.001 (***).

To investigate whether geographic origin influences biogenetic variations, altitude was used as the geographic parameter, and its correlation with the chromatic and odor-active components of LC and LX were examined. Hierarchical cluster analysis (HCA) based on Pearson correlation was conducted (Fig 8). The chromaticity parameters (ΔL*, Δa*, Δb*, and ΔE) exhibited strong intercorrelations (*p* < 0.001). For the electronic nose sensors, W1C showed significant positive correlations with W3C, W5C, W1S, W1W, W2S, W2W, and E3, but significant negative correlations with W3S, C1, and altitude (*p* < 0.001). Similarly, W5S correlated positively with W3C, W5C, W1S, W1W, and W2S, and negatively with W3S, C1, B3, and altitude (*p* < 0.001). W3C was positively associated with W5C, W1S, W1W, W2S, W2W, and E3, and negatively correlations with W3S, C1, and altitude (*p* < 0.001). W5C showed positive correlations with W1S, W1W, W2S, W2W, and E3, but negative correlations with W3S, C1, He, B3, and altitude (*p* < 0.001). W1S was positively correlated with W1W, W2S, W2W, and E3, and negatively with W3S, C1, B3, and altitude (*p* < 0.001). W1W exhibited positive correlations with W2S, W2W, and E3, and negative correlations with W3S, C1, B3, and altitude (*p* < 0.001). W2S correlated positively with W2W and E3, but negative with W3S, C1, B3, and altitude (*p* < 0.001). In contrast, W3S showed positive correlations with C1 and altitude (*p* < 0.001). Among the other chemical parameters, SI was positively correlated with SH, LI, 3B, and altitude (*p* < 0.001), while SH correlated positively correlated with LI and 3B (*p* < 0.001). LI also showed a significant positive correlation with 3B (*p* < 0.001). C1 was positively correlated with B3 and altitude, but negatively correlated with E3 (*p* < 0.001). Overall, altitude exhibited significant correlations with C1, B3, He, E3, and BH. Although the chromaticity parameters (ΔL*, Δa*, Δb*, ΔE) were strongly interrelated, their associations with most bioactive components were not statistically significant, suggesting that color changes may not predominantly originate from these active compounds. Notably, electronic nose sensor responses were significantly correlated with C1, B3, He, and E3, indicating a potential link between these constituents and the odor profiles of *Ligusticum chuanxiong* and related species.

## 4. Discussion and Conclusion

This study systematiclly compared collected LC and LX using intelligent sensory technology, HPLC, and HS-SPME-GC-MS, revealing significant quality differences. Morphologically, LX exhibited an irregular nodular cylindrical structure with branched rhizomes, prominent internodes, conspicuous annular protrusions, dense adventitious roots, and hollow stem bases connected to short apical branches, accompanied by indistinct cambium rings. In contrast, LC presented a fist-shaped clustered structure with unbranched rhizomes, parallel raised nodes, sparse adventitious roots, sunken stem scars without short branches, and well-defined cambium rings. Quantitative measurements showed that LX had significantly higher values in dry weight, length, and length-to-width ratio than LC. Chromaticity analysis indicated that LX powder appeared brown, whereas LC powder was yellowish-brown. Odor analysis demonstrated that LC exhibited stronger responses to the W5C, W1S, W2S and W1C sensors. Chemical composition analysis revealed that LX contained higher levels of SA but lower levels of the bioactive compounds LI, SI, SH, and 3B compared with LC; HS-SPME-GC-MS identified 110 volatile components—34 were shared, whereas LX and LC possessed 48 and 28 unique components, respectively. OPLS-DA modeling further screened 11 differential compounds. Correlation analysis showed significant associations between odor profiles and altitude, C1, B3, He, and E3 parameters.

Previous studies have reported that SI and SH have blood circulation-promoting effects. LI, FA and SI exhibit pronounced efficacy in regulating menstruation and alleviating pain [26,27]. In addition, SA has been shown to attenuates L-glutamate-induced neuronal damage and exerts neuroprotective effects [28,29]. In our study, the contents of SI, SH and LI were higher in LC than in LX, whereas FA and SA were more abundant in LX. These differences in chemical composition suggest that LC and LX may possess distinct bioactive potential, which warrants further comparative pharmacological investigations.

Previous research have suggested that environmental factors, particularly altitude, can influence the composition and accumulation of volatile oils and flavonoid [30,31]. For example, *Rhodiola crenulata* exhibits peak phenolic acid accumulation at mid-altitudes (3,800 m), whereas metabolic suppression occurs at extreme altitudes (>4,000 m) or under prolonged high-intensity UV-B radiation, indicating a threshold-dependent response to environmental stress. Similarly, *Angelica sinensis* exposed to low-temperature stress (15°C) shows a metabolic trade-off strategy in which ferulic acid content increases by 1.8–2.3 fold, while the activities of key enzymes involved in polysaccharide biosynthesis decrease by 40–55%, accompanied by reduced biomass accumulation [32,33]. In the present study, the altitudes of the sampled regions in Sichuan and Gansu were found to be significantly different. Altitude was positively correlated with C1, B3, He, E3 (*p* < 0.01). These findings provide insights for medicinal plant cultivation, suggesting that precise regulation of light intensity, temperature thresholds, and stress durations can help achieve a dynamic balance between secondary metabolite synthesis and plant growth vigor, thereby optimizing the yield and quality of active compounds.

Academician Huang Luqi proposed that the essence of genuine medicinal materials lies in “the same species in different locations”, implying that identical species adapt to diverse ecological environments and potentially undergoing genetic variations that contribute to distinct traits [34]. In other words, the phenotype of medicinal plants is determined by the combined effects of genetic regulation and environmental factors. In this study, LC and LX exhibited significant morphological differences. Correlation analysis further showed that altitude was positively associated with several chemical components (including C1, B3, 5P, He, and SA), suggesting that environmental conditions may contribute to the regulation of secondary metabolite accumulation. Notably, these compounds are key constituents of LC, and their accumulation appears to be influenced by ecological factors such as altitude. Historically, Gansu has been recognized as one of the principal genuine medicinal production regions for *L. chuanxiong.* From the perspective of the interactions between chemical composition and environmental factors, the high-altitude ecological conditions of Gansu may provide a material basis for the formation of the genuine quality characteristics of LC by promoting the synthesis and accumulation of specific secondary metabolites.

In conclusion, this study systematically characterized the differences between LX and LC, offering practical implications for enhancing the market competitiveness of LX, promoting the utilization of regional characteristic resources, and ensuring the sustainable development of the LX industry in Gansu Province. Nevertheless, certain limitations and future directions should be noted: (1) Expanding the sample size beyond the current 10 batches each from LX and LC and implementing reproducibility validation with robust statistical frameworks; (2) Integrating pharmacodynamic comparisons with chemical profiling to refine quality evaluation systems; (3) Applying metabolomics to elucidate biosynthetic pathways and regulatory mechanisms of significantly differential phthalides such as senkyunolides.

## Supporting information

S1 File Supplementary highlights. (DOCX)

S2 File Supplementary figures and tables. This file contains S1 Fig (electronic nose response curves for LC and LX), S2 Fig (mean maximum response values of electronic nose under different sample weights, particle sizes, incubation times, and injection volumes), S1 Table (sample sources), S2 Table (electronic nose metal oxide sensor information), S3 Table (standard curve regression equations), S4 Table (trait identification key points for LX and LC medicinal materials), S5 Table (similarity evaluation of fingerprints of LC and LX), S6 Table (eigenvalues and variance contribution rates), S7 Table (factor loading matrix), and S8 Table (volatile compounds detected in LC and LX by HS-GC-MS).
(DOCX)

S1 Fig Abstract. (TIF)
