# Supplementary material for: Comparative analysis of Ligusticum chuanxiong from Gansu and Sichuan using the fingerprint technique and HS-SPME-GC-MS combined with chemometric analysis
Source: PLoS One. 2026 Apr 30;21(4):e0347839. doi: 10.1371/journal.pone.0347839 (PMC13132178; doi:10.1371/journal.pone.0347839)
Supplement: S1 File — (DOCX) [file pone.0347839.s001.docx]

**Highlights**

- The HPLC fingerprint of *Ligusticum chuanxiong* (LC) and *Xixiong* (LX) was first established.
- Sixteen differential chemical components between LC and LX species were screened by chemometrics analysis.
- We clarified the differences in the appearance and odor chemical composition between LC and LX.
