# Supplementary material for: Comparative analysis of Ligusticum chuanxiong from Gansu and Sichuan using the fingerprint technique and HS-SPME-GC-MS combined with chemometric analysis
Source: PLoS One. 2026 Apr 30;21(4):e0347839. doi: 10.1371/journal.pone.0347839 (PMC13132178; doi:10.1371/journal.pone.0347839)
Supplement: S2 File — This file contains S1 Fig (electronic nose response curves for LC and LX), S2 Fig (mean maximum response values of electronic nose under different sample weights, particle sizes, incubation times, and injection volumes), S1 Table (sample sources), S2 Table (electronic nose metal oxide sensor information), S3 Table (standard curve regression equations), S4 Table (trait identification key points for LX and LC medicinal materials), S5 Table (similarity evaluation of fingerprints of LC and LX), S6 Table (eigenvalues and variance contribution rates), S7 Table (factor loading matrix), and S8 Table (volatile compounds detected in LC and LX by HS-GC-MS). (DOCX) [file pone.0347839.s002.docx]

**Supplementary Data**

**Abbreviations**

| Abbreviations | Full name |
| --- | --- |
| LC | Ligusticum chuanxiong |
| LX | Xixiong |
| HS-SPME-GC-MS | Headspace solid-phase microextraction-gas chromatography-mass spectrometry |
| HPLC | High performance liquid chromatography |
| TCM | Traditional Chinese Medicine |
| PCA | Principal component analysis |
| OPLS-DA | Orthogonal partial least squares-discriminant analysis |
| L* | Brightness |
| a* | Redness |
| b* | Yellowness |
| NF-κB | [Nuclear Factor-kappa B](https://www.baidu.com/s?ch=8&tn=25017023_2_dg&wd=Nuclear%20Factor-kappa%20B&usm=2&ie=utf-8&rsv_pq=82ae6053002c558f&oq=NF-%CE%BAB%E5%85%A8%E7%A7%B0&rsv_t=95a7xLtPldcoALI0Luz3hPr1edA+XWNjWs3gE5CBfrBfYZ7uls+fR9UZdDMrzT/K/gn3OA&sa=re_dqa_zy&icon=1) |
| TLR 4 | Toll Like Receptor 4 |
| SA | Senkyunolide A |
| SI | Senkyunolide I |
| SH | Senkyunolide H |
| 3B | 3-Butylidenephthalide |
| LI | Ligustilide |
| C1 | Cyclohexane, 1-methylene-4-(1-methylethenyl)- |
| E3 | (E)-3-Butylidene-4, 5-dihydroisobenzofuran-1(3H)-one |
| B3 | Bicyclo[3.1.1]heptane, 6, 6-dimethyl-2-methylene-(1S)- |
| 5P | 5-Pentylcyclohexa-1, 3-diene |
| Bh | Bicyclo[3.1.0]hexane, 4-methylene-1-(1-methylethyl)- |
| He | Hexadecanoicacid, methylester |
| Ph | Phenol, 5-ethenyl-2-methoxy- |
| 1R | (1R)-2, 6, 6-Trimethylbicyclo[3.1.1]hept-2-ene |
| Oc | 12, 15-Octadecadienoic acid, methyl ester |
| 2M | 2-Methoxy-4-vinylphenol |
| 2O | 2-Octanol |


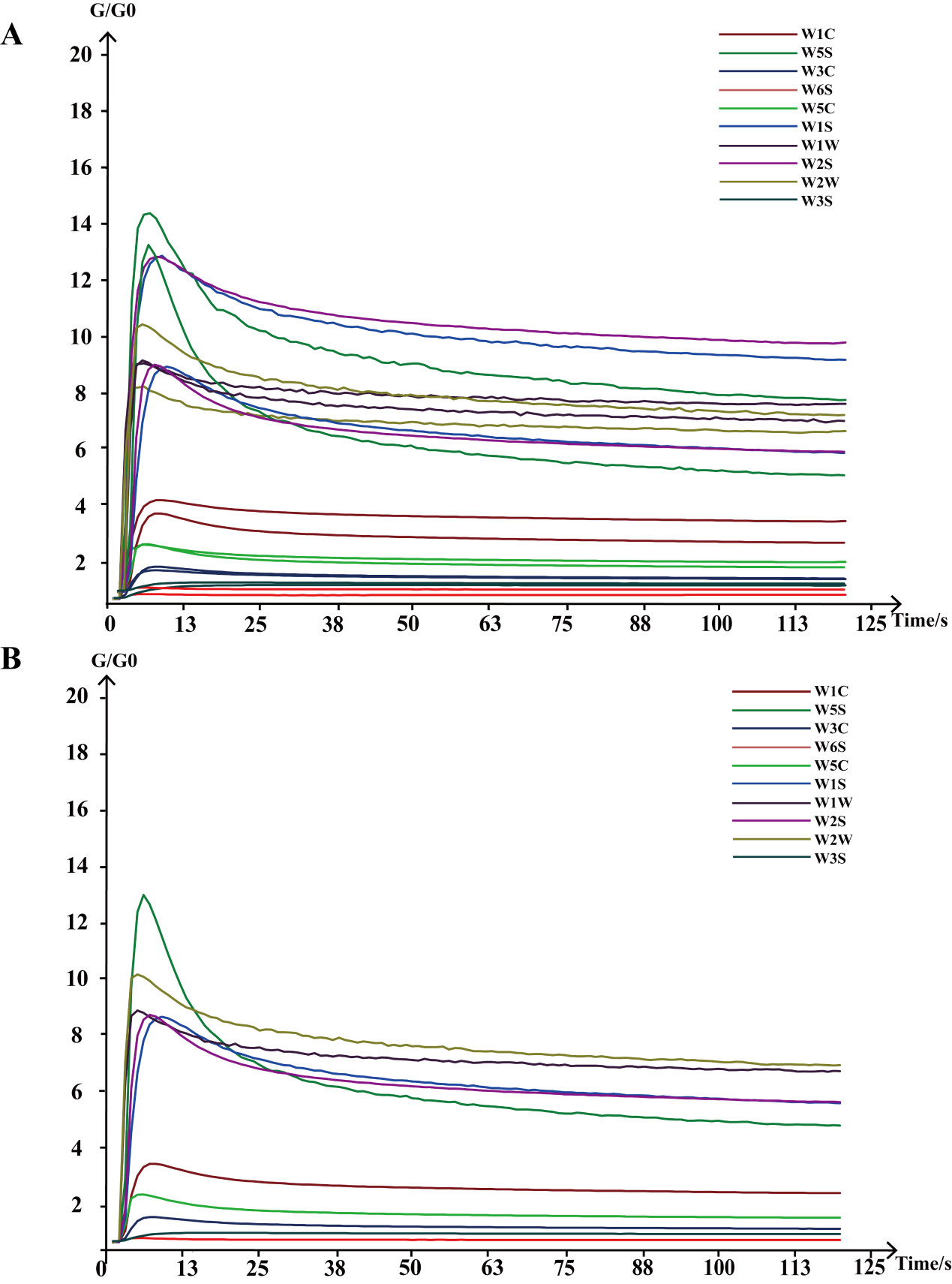


**S1 Fig.** (A) Response curve of electronic nose to LC; (B) Response curve of electronic nose to LX;


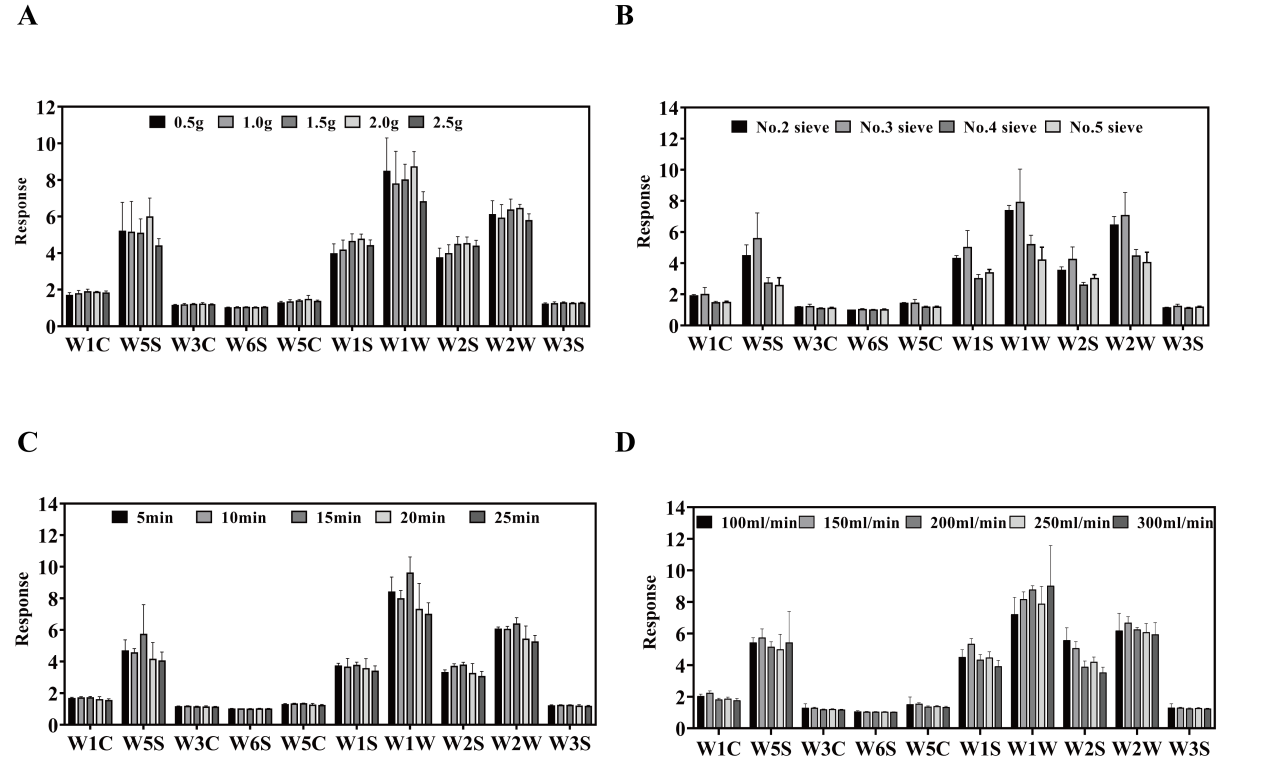


**S2 Fig.** (A, n=3) The mean value of the maximum response value of the sensor corresponding to different sample weights; (B, n=3) The mean value of the maximum response value of electronic noses with different particle sizes; (C, n=3) The mean value of the maximum response value of the electronic nose at different incubation times; (D, n=3) The mean response values of electronic noses with different injection volumes.

**S1 Table Sample source**

| Medicinal material label | Habitat | Harvest time | Altitude / m |
| --- | --- | --- | --- |
| LC1 | Jianfeng Town, Leshan City, Sichuan Province | 2023.4.27 | 434 |
| LC2 | Jiajiang County, Leshan City, Sichuan Province | 2023.4.27 | 416 |
| LC3 | Aoping Town, Pengzhou City, Sichuan Province | 2023.4.27 | 609 |
| LC4 | Yongfeng Village, Meishan, Sichuan Province | 2023.4.27 | 423 |
| LC5 | Yinfeng Town, Shifang City, Sichuan Province | 2023.4.27 | 540 |
| LC6 | Majing Town, Shifang City, Sichuan Province | 2023.4.27 | 515 |
| LC7 | Xinshi Town, Mianzhu City, Sichuan Province | 2023.4.28 | 555 |
| LC8 | Gongyi Town, Pengshan County, Meishan City, Sichuan Province | 2023.4.27 | 442 |
| LC9 | Hongyan Town, Pengzhou City, Sichuan Province | 2023.4.28 | 584 |
| LC10 | Xinglong Town, Mianzhu City, Sichuan Province | 2023.4.28 | 624 |
| LX1 | Shanzhai Township, Huating City, Pingliang City, Gansu Province | 2023.10.21 | 1900 |
| LX2 | Maxia Town, Huating City, Pingliang City, Gansu Province | 2023.10.21 | 1634 |
| LX3 | Daling Village, Maxia Town, Huating City, Pingliang City, Gansu Province | 2023.10.21 | 1910 |
| LX4 | Maxia Town Chechanggou Village, Huating City, Pingliang City, Gansu Province | 2023.10.22 | 2142 |
| LX5 | A hundred acres of Chinese herbal medicine planting base in Hexi Town, Huating River, Pingliang City, Gansu Province | 2023.10.22 | 1611 |
| LX6 | Huating Cedi Town Kongjiazhuang, Pingliang City, Gansu Province | 2023.10.22 | 1563 |
| LX7 | Xiyuan Village, Huating Xihua Town, Pingliang City, Gansu Province | 2023.10.22 | 1653 |
| LX8 | Shangguan Moping, Huating City, Pingliang City, Gansu Province | 2023.10.22 | 1697 |
| LX9 | Shanzhaixiatan Village, Huating City, Pingliang City, Gansu Province | 2023.10.22 | 1717 |
| LX10 | Shanzhai East Street Village, Huating City, Pingliang City, Gansu Province | 2023.10.22 | 1760 |

**S2 Table Electronic nose metal oxide sensor information**

| Array serial number | Sensor name | Performance profile |
| --- | --- | --- |
| 1 | W1C | aromatic |
| 2 | W5S | broadrange |
| 3 | W3C | aromatic |
| 4 | W6S | hydrogen |
| 5 | W5C | arom-aliph |
| 6 | W1S | broad-methane |
| 7 | W1W | sulphur-organic |
| 8 | W2S | broad-alcohol |
| 9 | W2W | sulph-chlor |
| 10 | W3S | methane-aliph |

**S3 Table Regression equation of standard curve**

| Proof sample | Standard curve | R2 | Range of linearity/（mg/ml） |
| --- | --- | --- | --- |
| Ferulic acid | y = 4×10^7^x - 203813 | 0.9993 | 0.0736~0.7360 |
| Senkyunolide I | y = 3×10^7^x + 46830 | 0.9999 | 0.0788~0.7880 |
| Senkyunolide H | y = 5×10^7^x - 30923 | 0.9994 | 0.00338~0.03379 |
| Senkyunolide A | y = 9×10^6^x - 379457 | 0.9995 | 0.7267~7.2673 |
| Ligustilide | y = 8×10^6^x + 545895 | 0.9996 | 0.5680~5.6800 |
| 3-Butylidenephthalide | y = 4×10^6^x + 118633 | 0.9995 | 0.3375~3.3750 |

**S4 Table Key Points for the Trait Identification of LX and LC Medicinal Materials**

|  | LX | LC |
| --- | --- | --- |
| Morphology | Irregular nodular cylindrical or clustered masses, rarely fist-shaped.。The rhizome is branched and the internodes are distinct。Surface exhibits irregular longitudinal furrows or raised annular nodes，Numerous adventitious roots at nodes, leaving multiple root scars.Apex bears several circular, hollow stem bases. | Irregular nodular fist-shaped clustered masses. Unbranched rhizome. Surface rough and wrinkled, with multiple parallel raised annulations. Apex displays a sunken, subcircular stem scar; small tubercle-like root scars beneath and on annulations. |
| Color | Brown | Yellowish-brown |
| Texture | Hard | Solid, difficult to fracture |
| Transverse section | Wood (xylem) pale yellow; cortex yellowish-white with numerous fissures and brown oil glands. Cambium ring indistinct. | Wood (xylem) yellowish-white or grayish-yellow, scattered with yellowish-brown oil cavities. Cambium ring wavy. |
| Odor and taste | Aromatic odor; bitter and pungent taste. | Intensely aromatic odor; bitter and pungent taste with a slight numbing sensation and subtle sweet aftertaste. |

**S5 Table Similarity evaluation of fingerprints of LC and LX**

|  | LC1 | LC2 | LC3 | LC4 | LC5 | LC6 | LC7 | LC8 | LC9 | LC10 | LX1 | LX2 | LX3 | LX4 | LX5 | LX6 | LX7 | LX8 | LX9 | LX10 |
| --- | --- | --- | --- | --- | --- | --- | --- | --- | --- | --- | --- | --- | --- | --- | --- | --- | --- | --- | --- | --- |
| LC1 | 1 | 0.98 | 0.986 | 0.98 | 0.988 | 0.988 | 0.969 | 0.984 | 0.983 | 0.985 | 0.717 | 0.706 | 0.721 | 0.87 | 0.635 | 0.69 | 0.833 | 0.758 | 0.693 | 0.921 |
| LC2 | 0.98 | 1 | 0.97 | 0.989 | 0.993 | 0.995 | 0.993 | 0.997 | 0.995 | 0.993 | 0.767 | 0.754 | 0.773 | 0.888 | 0.68 | 0.744 | 0.88 | 0.807 | 0.756 | 0.938 |
| LC3 | 0.986 | 0.97 | 1 | 0.99 | 0.967 | 0.968 | 0.977 | 0.981 | 0.97 | 0.965 | 0.785 | 0.774 | 0.792 | 0.903 | 0.706 | 0.767 | 0.885 | 0.821 | 0.765 | 0.942 |
| LC4 | 0.98 | 0.989 | 0.99 | 1 | 0.978 | 0.981 | 0.994 | 0.994 | 0.986 | 0.978 | 0.807 | 0.794 | 0.813 | 0.915 | 0.723 | 0.788 | 0.907 | 0.842 | 0.791 | 0.954 |
| LC5 | 0.988 | 0.993 | 0.967 | 0.978 | 1 | 0.998 | 0.978 | 0.992 | 0.993 | 0.998 | 0.703 | 0.69 | 0.703 | 0.851 | 0.612 | 0.672 | 0.825 | 0.745 | 0.685 | 0.912 |
| LC6 | 0.988 | 0.995 | 0.968 | 0.981 | 0.998 | 1 | 0.982 | 0.995 | 0.998 | 1 | 0.723 | 0.71 | 0.722 | 0.856 | 0.629 | 0.691 | 0.84 | 0.763 | 0.703 | 0.922 |
| LC7 | 0.969 | 0.993 | 0.977 | 0.994 | 0.978 | 0.982 | 1 | 0.994 | 0.988 | 0.979 | 0.813 | 0.799 | 0.819 | 0.909 | 0.725 | 0.794 | 0.914 | 0.847 | 0.802 | 0.953 |
| LC8 | 0.984 | 0.997 | 0.981 | 0.994 | 0.992 | 0.995 | 0.994 | 1 | 0.997 | 0.994 | 0.773 | 0.76 | 0.773 | 0.885 | 0.681 | 0.746 | 0.88 | 0.809 | 0.755 | 0.943 |
| LC9 | 0.983 | 0.995 | 0.97 | 0.986 | 0.993 | 0.998 | 0.988 | 0.997 | 1 | 0.997 | 0.759 | 0.745 | 0.754 | 0.872 | 0.663 | 0.725 | 0.866 | 0.794 | 0.736 | 0.939 |
| LC10 | 0.985 | 0.993 | 0.965 | 0.978 | 0.998 | 1 | 0.979 | 0.994 | 0.997 | 1 | 0.716 | 0.703 | 0.712 | 0.847 | 0.62 | 0.681 | 0.833 | 0.755 | 0.694 | 0.919 |
| LX1 | 0.717 | 0.767 | 0.785 | 0.807 | 0.703 | 0.723 | 0.813 | 0.773 | 0.759 | 0.716 | 1 | 0.999 | 0.991 | 0.928 | 0.98 | 0.991 | 0.974 | 0.996 | 0.993 | 0.924 |
| LX2 | 0.706 | 0.754 | 0.774 | 0.794 | 0.69 | 0.71 | 0.799 | 0.76 | 0.745 | 0.703 | 0.999 | 1 | 0.988 | 0.927 | 0.986 | 0.988 | 0.966 | 0.994 | 0.991 | 0.92 |
| LX3 | 0.721 | 0.773 | 0.792 | 0.813 | 0.703 | 0.722 | 0.819 | 0.773 | 0.754 | 0.712 | 0.991 | 0.988 | 1 | 0.939 | 0.972 | 0.998 | 0.981 | 0.995 | 0.996 | 0.912 |
| LX4 | 0.87 | 0.888 | 0.903 | 0.915 | 0.851 | 0.856 | 0.909 | 0.885 | 0.872 | 0.847 | 0.928 | 0.927 | 0.939 | 1 | 0.911 | 0.924 | 0.964 | 0.952 | 0.925 | 0.966 |
| LX5 | 0.695 | 0.68 | 0.706 | 0.723 | 0.612 | 0.629 | 0.725 | 0.681 | 0.663 | 0.62 | 0.98 | 0.986 | 0.972 | 0.911 | 1 | 0.976 | 0.929 | 0.974 | 0.979 | 0.924 |
| LX6 | 0.69 | 0.744 | 0.767 | 0.788 | 0.672 | 0.691 | 0.794 | 0.746 | 0.725 | 0.681 | 0.991 | 0.988 | 0.998 | 0.924 | 0.976 | 1 | 0.972 | 0.992 | 0.997 | 0.914 |
| LX7 | 0.833 | 0.88 | 0.885 | 0.907 | 0.825 | 0.84 | 0.914 | 0.88 | 0.866 | 0.833 | 0.974 | 0.966 | 0.981 | 0.964 | 0.929 | 0.972 | 1 | 0.988 | 0.973 | 0.964 |
| LX8 | 0.758 | 0.807 | 0.821 | 0.842 | 0.745 | 0.763 | 0.847 | 0.809 | 0.794 | 0.755 | 0.996 | 0.994 | 0.995 | 0.952 | 0.974 | 0.992 | 0.988 | 1 | 0.994 | 0.942 |
| LX9 | 0.693 | 0.756 | 0.765 | 0.791 | 0.685 | 0.703 | 0.802 | 0.755 | 0.736 | 0.694 | 0.993 | 0.991 | 0.996 | 0.925 | 0.979 | 0.997 | 0.973 | 0.994 | 1 | 0.903 |
| LX10 | 0.921 | 0.938 | 0.942 | 0.954 | 0.912 | 0.922 | 0.953 | 0.943 | 0.939 | 0.919 | 0.924 | 0.92 | 0.912 | 0.966 | 0.974 | 0.941 | 0.964 | 0.942 | 0.903 | 1 |

**S6 Table Eigenvalues and Variance Contribution Rates**

| Principal component factors | characteristic value | Variance contribution rate% | Cumulative variance contribution rate% |
| --- | --- | --- | --- |
| 1 | 8.911 | 42.434 | 42.434 |
| 2 | 5.645 | 26.881 | 69.315 |
| 3 | 2.819 | 13.426 | 82.741 |
| 4 | 1.03 | 4.904 | 87.644 |

**S7 Table Factor loading matrix**

|  | 1 | 2 | 3 | 4 |
| --- | --- | --- | --- | --- |
| peak 1 | 0.313 | -0.038 | 0.884 | -0.021 |
| peak 2 | -0.239 | -0.106 | 0.683 | -0.265 |
| peak 3 | 0.652 | -0.054 | 0.303 | -0.264 |
| peak 4 | 0.567 | 0.233 | 0.624 | 0.29 |
| peak 5 | 0.07 | 0.799 | -0.419 | 0.167 |
| peak 6 | 0.393 | 0.845 | 0.241 | -0.023 |
| peak 7 | 0.091 | 0.965 | 0.134 | 0.042 |
| peak 8 | 0.824 | 0.197 | 0 | -0.191 |
| peak 9 | 0.962 | 0.179 | 0.042 | 0.139 |
| peak 10 | 0.963 | 0.185 | 0.055 | 0.126 |
| peak 11 | 0.958 | 0.064 | -0.006 | 0.003 |
| peak 12 | 0.91 | 0.292 | 0.029 | 0.112 |
| peak 13 | 0.166 | 0.967 | 0.021 | 0.053 |
| peak 14 | 0.064 | 0.984 | -0.008 | 0.137 |
| peak 15 | -0.616 | 0.64 | 0.266 | -0.087 |
| peak 16 | 0.292 | 0.793 | -0.178 | 0.359 |
| peak 17 | -0.577 | 0.455 | 0.649 | 0.04 |
| peak 18 | 0.071 | 0.23 | -0.116 | 0.875 |
| peak 19 | -0.758 | 0.476 | 0.337 | -0.13 |
| peak 20 | 0.828 | 0.168 | -0.321 | 0.115 |
| peak 21 | 0.869 | 0.179 | 0.237 | 0.291 |

**S8 Table Detection of volatile compounds in LC and LX by HS-**SPME-**GC-MS**

| Numbering | Compound | Molecular formula | CAS | Comparative content(%) | | | |
| --- | --- | --- | --- | --- | --- | --- | --- |
|  |  |  |  | LC | LX | |  |
| 1 | Z-Butylidenephthalide | C_12_H_12_O_2_ | 72917-31-8 | 1.964 | | 0.625 | |
| 2 | (Z)-3-Butylidene-4,5-dihydroisobenzofuran-1(3H)-one | C_12_H_14_O_2_ | 81944-09-4 | 0.642 | | 4.636 | |
| 3 | (3S,3aR)-3-Butyl-3a,4,5,6-tetrahydroisobenzofuran-1(3H)-one | C_12_H_18_O_2_ | 4567-33-3 | 1.852 | | 4.686 | |
| 4 | Senkyunolide | C_12_H_16_O_2_ | 63038-10-8 | 18.190 | | 36.027 | |
| 5 | 3-Butylisobenzofuran-1(3H)-one | C_12_H_14_O_2_ | 6066-49-5 | 1.906 | | 3.641 | |
| 6 | 3-Cyclohexen-1-ol, 4-methyl-1-(1-methylethyl)-, (R)- | C_10_H_18_O | 20126-76-5 | 0.206 | | - | |
| 7 | 1H-Cycloprop[e]azulen-7-ol,decahydro-1,1,7-trimethyl-4-methylene-,[1ar-(1a.alpha.,4a.alpha.,7.beta.,7a.beta.,7b.alpha.)- | C_15_H_24_O | 6750-60-3 | 0.381 | | - | |
| 8 | Benzenemethanol, .alpha.,.alpha.,4-trimethyl- | C_10_H_14_O | 1197-01-9 | 0.044 | | - | |
| 9 | 2-Octanol | C_8_H_18_O | 123-96-6 | / | | / | |
| 10 | 3,4-Dimethylbenzyl alcohol | C_9_H_12_O | 6966-10-5 | 0.080 | | - | |
| 11 | Linalool | C_10_H_18_O | 78-70-6 | - | | 0.108 | |
| 12 | .alpha.-Bisabolol | C_15_H_26_O | 515-69-5 | - | | 0.573 | |
| 13 | Cyclododecanol | C_12_H_24_O | 1724-39-6 | - | | 0.097 | |
| 14 | Isospathulenol | C_15_H_24_O | 88395-46-4 | 0.229 | | - | |
| 15 | Cubenene | C_15_H_24_ | 29837-12-5 | 0.281 | | - | |
| 16 | Naphthalene,1,2,3,4,4a,5,6,8a-octahydro-7-methyl-4-methylene-1-(1-methylethyl)-,(1.alpha.,4a.beta.,8a.alpha.)- | C_15_H_24_ | 39029-41-9 | 0.097 | | 0.162 | |
| 17 | Naphthalene,1,2,4a,5,8,8a-hexahydro-4,7-dimethyl-1-(1-methylethyl)-,[1S-(1.alpha.,4a.beta.,8a.alpha.)]- | C_15_H_24_ | 523-47-7 | - | | 0.269 | |
| 18 | Benzene, 1-(1,5-dimethylhexyl)-4-methyl- | C_15_H_24_ | 1461-02-5 | 0.197 | | - | |
| 19 | Naphthalene, 1,2,3,4,4a,7-hexahydro-1,6-dimethyl-4-(1-methylethyl)- | C_15_H_24_ | 16728-99-7 | 0.218 | | - | |
| 20 | (1R,3aR,4aR,8aR)-1,4,4,6-Tetramethyl-1,2,3,3a,4,4a,7,8-octahydrocyclopenta[1,4]cyclobuta[1,2]benzene | C_15_H_24_ | 94482-89-0 | - | | 0.214 | |
| 21 | (1S,4S,4aS)-1-Isopropyl-4,7-dimethyl-1,2,3,4,4a,5-hexahydronaphthalene | C_15_H_24_ | 267665-20-3 | - | | 0.172 | |
| 22 | 1-Phenyl-1-decanol | C_16_H_26_O | 21078-95-5 | - | | 15.238 | |
| 23 | 1-Isopropyl-4,7-dimethyl-1,2,3,5,6,8a-hexahydronaphthalene | C_15_H_24_ | 16729-01-4 | - | | 0.179 | |
| 24 | p-Cymene | C_10_H_14_ | 99-87-6 | 0.167 | | 0.151 | |
| 25 | Benzene, 1-methyl-3-(1-methylethyl)- | C_10_H_14_ | 535-77-3 | 0.163 | | 0.173 | |
| 26 | o-Cymene | C_10_H_14_ | 527-84-4 | 0.190 | | 0.185 | |
| 27 | (-)-Spathulenol | C_15_H_24_O | 77171-55-2 | 0.224 | | - | |
| 28 | 2-Methoxy-4-vinylphenol | C_9_H_10_O_2_ | 7786-61-0 | 0.148 | | 0.080 | |
| 29 | Phenol, 5-ethenyl-2-methoxy- | C_9_H_10_O_2_ | 621-58-9 | 0.118 | | 0.114 | |
| 30 | p-Cresol | C_7_H_8_O | 106-44-5 | - | | 0.136 | |
| 31 | Methyleugenol | C_11_H_14_O_2_ | 93-15-2 | - | | 0.091 | |
| 32 | Phenol, 2-methyl- | C_7_H_8_O | 95-48-7 | - | | 0.118 | |
| 33 | 1,2-Ethanediol, 1,2-diphenyl-, [R-(R*,R*)]- | C_14_H_14_O_2_ | 52340-78-0 | - | | 0.045 | |
| 34 | 2,5-Dimethylanisole | C_9_H_12_O | 1706-11-2 | - | | 0.090 | |
| 35 | Benzene, 2-methoxy-1,3-dimethyl- | C_9_H_12_O | 1004-66-6 | - | | 0.113 | |
| 36 | 3,4-Dimethylanisole | C_9_H_12_O | 4685-47-6 | - | | 0.051 | |
| 37 | 1H-Cyclopropa[a]naphthalene,decahydro-1,1,3a-trimethyl-7-methylene-,[1aS-(1a.alpha.,3a.alpha.,7a.beta.,7b.alpha.)]- | C_15_H_24_ | 112-54-9 | - | | 0.119 | |
| 38 | Dodecanal | C_12_H_24_O | 66-25-1 | - | | 0.122 | |
| 39 | Hexanal | C_6_H_12_O | 465-28-1 | 0.027 | | - | |
| 40 | Carotol | C_15_H_26_O | 81944-08-3 | - | | 0.130 | |
| 41 | (E)-3-Butylidene-4,5-dihydroisobenzofuran-1(3H)-one | C_12_H_14_O_2_ | 119-53-9 | 39.967 | | 20.240 | |
| 42 | Benzoin | C_14_H_12_O_2_ | 1450-72-2 | 0.136 | | - | |
| 43 | Ethanone, 1-(2-hydroxy-5-methylphenyl)- | C9H10O2 | 128575-99-5 | - | | 0.108 | |
| 44 | 3-Pentyl-4,5-dihydroisobenzofuran-1(3H)-one | C_13_H_18_O_2_ | 875-59-2 | 0.122 | | - | |
| 45 | 4-Hydroxy-2-methylacetophenone | C_9_H_10_O_2_ | 1009-14-9 | - | | 0.084 | |
| 46 | 1-Pentanone, 1-phenyl- | C_11_H_14_O | 488-97-1 | 0.048 | | - | |
| 47 | Tricyclo[2.2.1.0(2,6)]heptane, 1,3,3-trimethyl- | C_10_H_16_ | 499-97-8 | 0.119 | | 0.393 | |
| 48 | Cyclohexane, 1-methylene-4-(1-methylethenyl)- | C_10_H_16_ | 636-41-9 | 0.088 | | 0.356 | |
| 49 | 1H-Pyrrole, 2-methyl- | C_5_H_7_N | 242794-76-9 | 0.603 | | - | |
| 50 | Bicyclo[5.2.0]nonane,2-methylene-4,8,8-trimethyl-4-vinyl- | C_15_H_24_ | 3387-41-5 | - | | 0.187 | |
| 51 | Bicyclo[3.1.0]hexane,4-methylene-1-(1-methylethyl)- | C_10_H_16_ | 20071-49-2 | 0.742 | | 0.284 | |
| 52 | isoledene | C_15_H_24_ | 95910-36-4 | - | | 0.159 | |
| 53 | (+)-4-Carene | C_10_H_16_ | 29050-33-7 | 0.460 | | 0.220 | |
| 54 | (1R,5R)-2-Methyl-5-((R)-6-methylhept-5-en-2-yl)bicyclo[3.1.0]hex-2-ene | C_15_H_24_ | 58319-06-5 | - | | 0.166 | |
| 55 | (1S,2E,6E,10R)-3,7,11,11-Tetramethylbicyclo[8.1.0]undeca-2,6-diene | C_15_H_24_ | 24703-35-3 | 0.416 | | - | |
| 56 | Cyclohexene,4-ethenyl-4-methyl-3-(1-methylethenyl)-1-(1-methylethyl)-, (3R-trans)- | C_15_H_24_ | 20307-84-0 | 0.664 | |  | |
| 57 | cis-.beta.-Farnesene | C_15_H_24_ | 28973-97-9 | - | | 0.149 | |
| 58 | (E)-.beta.-Famesene | C_15_H_24_ | 18794-84-8 | - | | 0.140 | |
| 59 | Azulene,1,2,3,3a,4,5,6,7-octahydro-1,4-dimethyl-7-(1-methylethenyl)-,[1R-(1.alpha.,3a.beta.,4.alpha.,7.beta.)]- | C_15_H_24_ | 22567-17-5 | 0.918 | | - | |
| 60 | 1,5-Cyclodecadiene,1,5-dimethyl-8-(1-methylethenyl)-, [S-(Z,E)]- | C_15_H_24_ | 75023-40-4 | - | | 0.177 | |
| 61 | Cyclohexene, 1-butyl- | C_10_H_18_ | 3282-53-9 | 0.045 | | 0.080 | |
| 62 | 1H-Benzocycloheptene,2,4a,5,6,7,8-hexahydro-3,5,5,9-tetramethyl-, (R)- | C_15_H_24_ | 1461-03-6 | - | | 0.228 | |
| 63 | Bicyclo[3.1.0]hex-2-ene,2-methyl-5-(1-methylethyl)- | C_10_H_16_ | 2867-05-2 | 0.138 | | 0.135 | |
| 64 | 2,4,6-Octatriene, 3,4-dimethyl- | C_10_H_16_ | 57396-75-5 | - | | 0.194 | |
| 65 | Bicyclo[3.1.1]hept-2-ene, 3,6,6-trimethyl- | C_10_H_16_ | 4889-83-2 | - | | 0.348 | |
| 66 | .alpha.-Farnesene | C_15_H_24_ | 502-61-4 | - | | 0.196 | |
| 67 | Cyclohexene, 3-methyl-6-(1-methylethylidene)- | C_10_H_16_ | 586-63-0 | - | | 0.267 | |
| 68 | 3-Carene | C_10_H_16_ | 13466-78-9 | 0.167 | | 0.108 | |
| 69 | .beta.-Phellandrene | C_10_H_16_ | 555-10-2 | 0.387 | | 0.201 | |
| 70 | Bicyclo[3.1.0]hex-2-ene, 4,4,6,6-tetramethyl- | C_10_H_16_ | 19487-09-3 | - | | 0.109 | |
| 71 | Cyclohexene,4-[(1E)-1,5-dimethyl-1,4-hexadien-1-yl]-1-methyl- | C_15_H_24_ | 25532-79-0 | - | | 0.109 | |
| 72 | Bicyclo[3.1.0]hex-2-ene,4-methyl-1-(1-methylethyl)- | C_10_H_16_ | 28634-89-1 | 0.096 | | 0.126 | |
| 73 | Terpinen-4-ol | C_10_H_18_O | 562-74-3 | 0.273 | | - | |
| 74 | 5-Pentylcyclohexa-1,3-diene | C_11_H_18_ | 56318-84-4 | 0.626 | | 1.148 | |
| 75 | 6-Butyl-1,4-cycloheptadiene | C_11_H_18_ | 22735-58-6 | 1.003 | | - | |
| 76 | .beta.-Pinene | C_10_H_16_ | 127-91-3 | 0.103 | | 0.196 | |
| 77 | (E,E)-1,3,5-Undecatriene | C_11_H_18_ | 19883-29-5 | - | | 0.138 | |
| 78 | 1H-Cycloprop[e]azulen-7-ol,decahydro-1,1,7-trimethyl-4-methylene-,[1ar-(1a.alpha.,4a.alpha.,7.beta.,7a.beta.,7b.alpha.)]- | C_15_H_24_O | 6750-60-3 | 0.325 | | - | |
| 79 | .alpha.-Guaiene | C_15_H_24_ | 3691-12-1 | 0.681 | | - | |
| 80 | Cyclohexane,1-ethenyl-1-methyl-2,4-bis(1-methylehenyl)-, [1S-(1.alpha.,2.beta.,4.beta.)]- | C_15_H_24_ | 515-13-9 | 0.132 | | - | |
| 81 | Naphthalene,decahydro-4a-methyl-1-methylene-7-(1-methylethenyl)-,[4aR-(4a.alpha.,7.alpha.,8a.beta.)]- | C_15_H_24_ | 17066-67-0 | 1.072 | | - | |
| 82 | Caryophyllene | C_15_H_24_ | 87-44-5 |  | | 0.192 | |
| 83 | .gamma.-Terpinene | C_10_H_16_ | 99-85-4 | 0.352 | | 0.342 | |
| 84 | Naphthalene,1,2,3,5,6,8a-hexahydro-4,7-dimethyl-1-(1-methylethyl)-, (1S-cis)- | C_15_H_24_ | 483-76-1 | - | | 0.258 | |
| 85 | Naphthalene,1,2,3,5,6,7,8,8a-octahydro-1,8a-dimethyl-7-(1-methylethenyl)-,[1R-(1.alpha.,7.beta.,8a.alpha.)]- | C_15_H_24_ | 4630-07-3 | 1.327 | | 0.111 | |
| 86 | 1,5-Cyclodecadiene,1,5-dimethyl-8-(1-methylethylidene)-, (E,E)- | C_15_H_24_ | 15423-57-1 | 0.128 | | - | |
| 87 | trans-.alpha.-Bergamotene | C_15_H_24_ | 13474-59-4 | - | | 0.168 | |
| 88 | Germacrene D | C_15_H_24_ | 23986-74-5 | - | | 0.214 | |
| 89 | (1R)-2,6,6-Trimethylbicyclo[3.1.1]hept-2-ene | C_10_H_16_ | 7785-70-8 | 0.116 | | 0.262 | |
| 90 | Bicyclosesquiphellandrene | C_15_H_24_ | 54324-03-7 | - | | 0.142 | |
| 91 | .alpha.-Phellandrene | C_10_H_16_ | 99-83-2 | 0.382 | | 0.153 | |
| 92 | cis-.alpha.-Bergamotene | C_15_H_24_ | 18252-46-5 | - | | 0.138 | |
| 93 | cis-Muurola-4(15),5-diene | C_15_H_24_ | 157477-72-0 | - | | 0.106 | |
| 94 | 1,3-Cyclohexadiene, 1-methyl-4-(1-methylethyl)- | C_10_H_16_ | 99-86-5 | 0.077 | | 0.158 | |
| 95 | Cyclohexene, 1-methyl-4-(1-methylethylidene)- | C_10_H_16_ | 586-62-9 | 0.401 | | 0.232 | |
| 96 | (1S)-(1)-beta-Pinene;(1S)-6,6-Dimethyl-2-methyleneb | C_10_H_16_ | 18172-67-3 | 0.067 | | 0.214 | |
| 97 | 5-Azulenemethanol,1,2,3,4,5,6,7,8-octahydro-.alpha.,.alpha.,3,8-tetramethyl-,acetate,[3S-(3.alpha.,5.alpha.,8.alpha.)]- | C_17_H_28_O_2_ | 54274-73-6 | - | | 0.074 | |
| 98 | 12,15-Octadecadienoic acid, methyl ester | C_19_H_34_O_2_ | 142878-08-8 | 0.181 | | 0.255 | |
| 99 | 3-(4-Methylbenzoyl)-2-thioxo-4-thiazolyl 4-methylbenzoate | C_19_H_15_NO_3_S_2_ | 4045-44-7 | - | | 0.063 | |
| 100 | 9,12-Octadecadienoic acid (Z,Z)-, methyl ester | C_19_H_34_O_2_ | 134-28-1 | 0.276 | | 0.262 | |
| 101 | Benzoic acid, 2-propenyl ester | C_10_H_10_O_2_ | 57156-97-5 | 0.048 | | - | |
| 102 | 1,5-Dimethyl-1-vinyl-4-hexenyl butyrate | C_14_H_24_O_2_ | 299929-13-8 | - | | 0.085 | |
| 103 | Tridecanoic acid, methyl ester | C_14_H_28_O_2_ | 112-63-0 | - | | 0.382 | |
| 104 | Acetic acid, decyl ester | C_12_H_24_O_2_ | 583-04-0 | - | | 0.105 | |
| 105 | Lauryl acetate | C_14_H_28_O_2_ | 78-36-4 | - | | 0.106 | |
| 106 | Hexadecanoic acid, methyl ester | C_17_H_34_O_2_ | 1731-88-0 | 0.169 | | 0.213 | |
| 107 | (+)-epi-Bicyclosesquiphellandrene | C_15_H_24_ | 112-17-4 | 0.059 | | 0.099 | |
| 108 | (3S,3aS,8aR)-6,8a-Dimethyl-3-(prop-1-en-2-yl)-1,2,3,3a,4,5,8,8a-octahydroazulene | C_15_H_24_ | 112-66-3 | 0.158 | | - | |
| 109 | 4-Acetoxy-3-methoxystyrene | C_11_H_12_O_3_ | 112-39-0 | 0.098 | | - | |
| 110 | 1,3-Cyclopentadiene, 1,2,3,4,5-pentamethyl- | C_10_H_16_ | 46316-15-8 | 0.055 | | - | |
