# Supplementary figures and images for: Comparative analysis of Ligusticum chuanxiong from Gansu and Sichuan using the fingerprint technique and HS-SPME-GC-MS combined with chemometric analysis
Source: PLoS One. 2026 Apr 30;21(4):e0347839. doi: 10.1371/journal.pone.0347839 (PMC13132178; doi:10.1371/journal.pone.0347839)

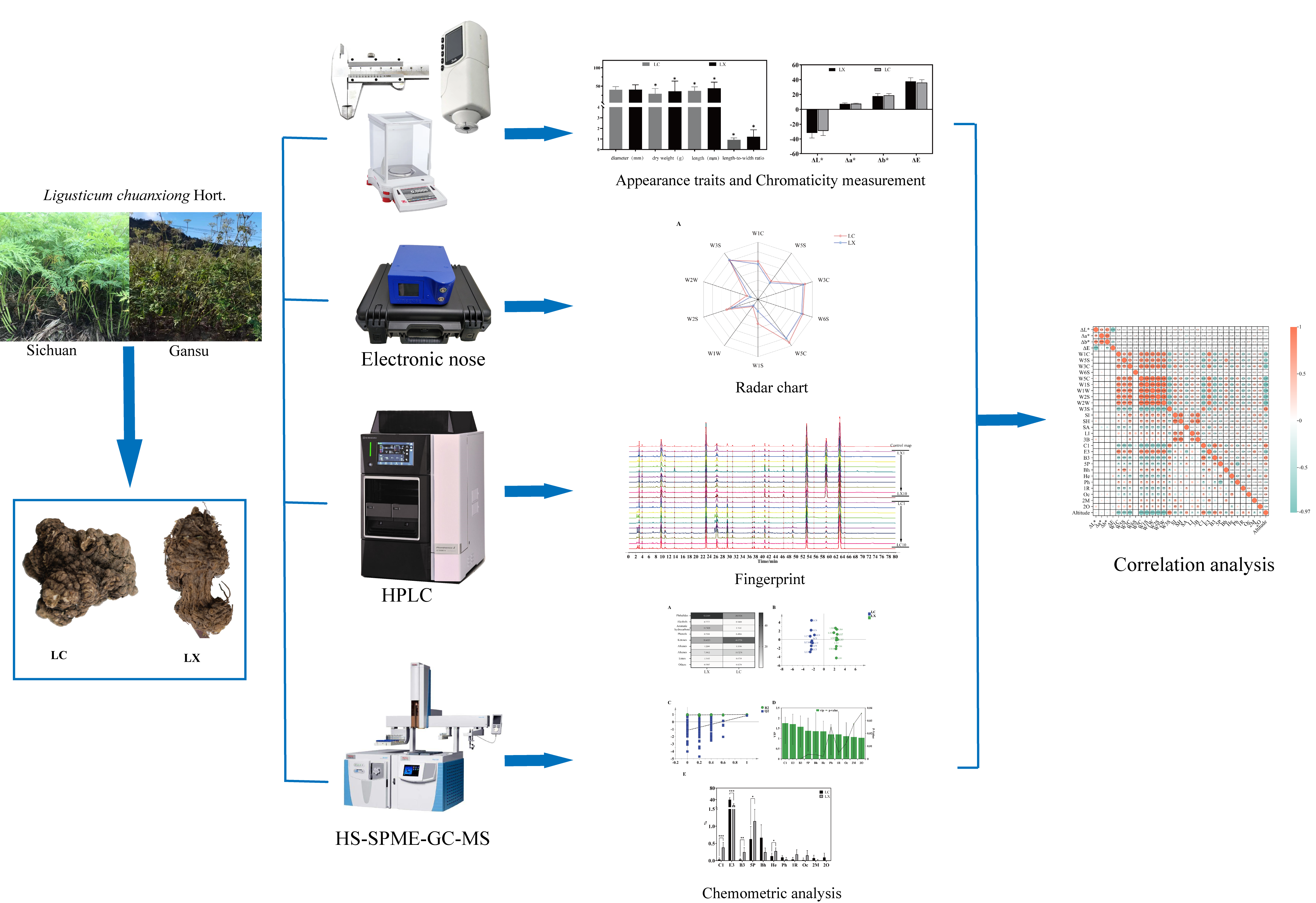

Supplement: S1 Fig — (TIF) [file pone.0347839.s003.tif]
